# What Characteristics of Urban Green Spaces and Recreational Activities Do Self-Reported Stressed Individuals Like? A Case Study of Baoji, China

**DOI:** 10.3390/ijerph16081348

**Published:** 2019-04-15

**Authors:** Tian Gao, Rui Song, Ling Zhu, Ling Qiu

**Affiliations:** College of Landscape Architecture and Arts, Northwest A&F University, Xianyang 712100, Shaanxi, China; tian.gao@nwsuaf.edu.cn (T.G.); songr@nwafu.edu.cn (R.S.); zhuling@nwafu.edu.cn (L.Z.)

**Keywords:** urban green space, human health, recreational activities, perceived sensory dimensions, landscape planning

## Abstract

Several studies have revealed the positive effects of green space and certain activities on counteracting the physical and mental pressure felt by human beings. However, how self-reported stressed people perceive restorative green spaces, their preferences for specific characteristics and activities, and what characteristics of urban green space can induce various types of activities for stress recovery has not been fully examined in the high-density cities of China. Using an on-site questionnaire survey conducted in the People’s Park (PP) in Baoji, China, this study is the first to consider the relationship among eight sensory dimensions, activity types and stress recovery in Chinese green space. Results showed that the highest-stressed respondents were more likely to spend their time in multi-layered woodlands adjacent to water, with more experiences of *serene* but less about *prospect*. They preferred *serene* dimension more, while the *culture* and *social* dimensions were least preferred. Sports and leisure activities and quiet activities were the most popular among highest-stressed respondents, which were positively related to the *serene* and *nature* dimensions, respectively. Results suggested that the most restorative environment for stress recovery can be regarded as multi-layered woodlands adjacent to water with more *serene* and *nature*, less *prospect* and few or no *culture* and *social* dimensions.

## 1. Introduction

The rapid development of urbanisation has led to the conversion of farmlands, grasslands, forests and other types of green spaces into newly urbanised areas [1], especially in developing countries [2]. As a result, urban life-related diseases such as mental stress due to less provision of green spaces have inevitably developed [3].

Stress, which is closely related to mental health [4] has become an important component of a healthy life for urban inhabitants. The World Health Organisation (WHO) clarified the definition of health in 1947: “Health is a state of complete physical, mental and social well-being and not merely the absence of disease or infirmity” [5]. Moderate pressure can motivate people when there is good harmonization between their daily life and environment [6]. Nevertheless, once needs and circumstances are not balanced, or even beyond the acceptance of the individual’s ability, this can lead to dissatisfaction with life and oneself, which then affects their normal life and creates pressure [7,8]. Undue pressure can easily give rise to a suppressed immune system [9] and aggressive cancer cells [10], even leading to premature death [11]. 

Hence, relevant stress studies have been extensively developed in many fields ranging from medicine to environmental psychology to landscape architecture. A series of stress evaluation methods and restoration theories have been put forward and widely applied in urban green space studies, such as Attention Restoration Theory (ART) [12], Psychophysiological Stress Recovery Theory (PSRT) [13] and Perceived Stress Scale (PSS) [14]. Several studies have shown that the natural environment was more beneficial to human health than the built environment [13,15,16], both physically and mentally [17,18]. Green space is an important component of the natural environment. It indirectly acts as a barrier between health and an illness lifestyle [19], and it plays a great role in stress relief in urban areas [20]. Exposure to green space can not only lower blood pressure, but it can also reduce stress levels [21,22]. The ability of green space to reduce stress is generally attributed to the fact that it provides city inhabitants with space for daily activities and social association according to its characteristics [23]. Certain activity types (e.g., [24,25]) can significantly reduce stress while consuming energy. WHO claimed that a lack of activities is one of the factors affecting physical and mental health [26].

However, due to the lack of knowledge on what characteristics of green space are associated with mental health restoration in urban environmental settings, it has become a great challenge for landscape architects to design urban green spaces that can better match the public’s needs for mental relief. A number of research studies have intended to classify characteristics of nature. One such classification system has been developed over the last 30 years by researchers at the Swedish University of Agricultural Sciences. The latest version distinguished the following eight perceived sensory dimensions: ‘*serene*’ (e.g., silent and calm), ‘*nature*’ (e.g., wild and untouched), ‘*rich in species*’ (e.g., many animals and plants), ‘*space*’ (e.g., spacious and free), ‘*prospect*’ (e.g., flat and well-cut lawns with scattered trees), ‘*refuge*’ (e.g., an enclosed and safe place), ‘*social*’ (e.g., entertainment and exhibitions) and ‘*culture*’ (e.g., decorated with fountains and ornamental plants) [27]. These eight perceived sensory dimensions have been increasingly applied in several studies and planning processes, as well as in policy documents, especially studies of the relationship between landscape characteristics and stress recovery [28]. Peschardt and Stigsdotter found that ‘*serene*’ and ‘*social*’ were the most significant attributes to the perceived recovery for people in small, public urban green spaces [29]. Memari et al. declared that an environment with more ‘*serene*’, ‘*nature*’ and ‘*refuge*’ dimensions and less ‘*social*’ and ‘*rich in species*’ dimensions can be beneficial to human mental restoration [30]. Several studies have also explored the association between the eight perceived sensory dimensions and corresponding recreational activities. Stigsdotter and Grahn found that rest activities could be connected with ‘*nature*’ [6]. Lottrup et al. showed that employees preferred ‘*serene*’ green spaces for rest and body activities, and ‘*refuge*’ and ‘*space*’ for sun-bathing activities in their work places [31]. Although certain relationships were identified in previous studies, the triangular associations among the eight perceived sensory dimensions, recreational activity and mental stress have not been fully examined in Chinese urban environmental settings. Therefore, in order to fill in this knowledge gap and further explore the associations between the perceived restorativeness of urban green spaces and the eight perceived sensory dimensions for the self-reported stressed individuals in China, this study’s specific objectives were to investigate:

1. Whether perceptions of the eight sensory dimensions differ between self-reported stressed user groups of urban green space in China.
2. The preferences of the self-reported stressed individuals for the eight sensory dimensions and specific recreational activities.
3. Correlations between the eight sensory dimensions and recreational activities.

## 2. Materials and Methods

### 2.1. Study Area

**Figure 1:**
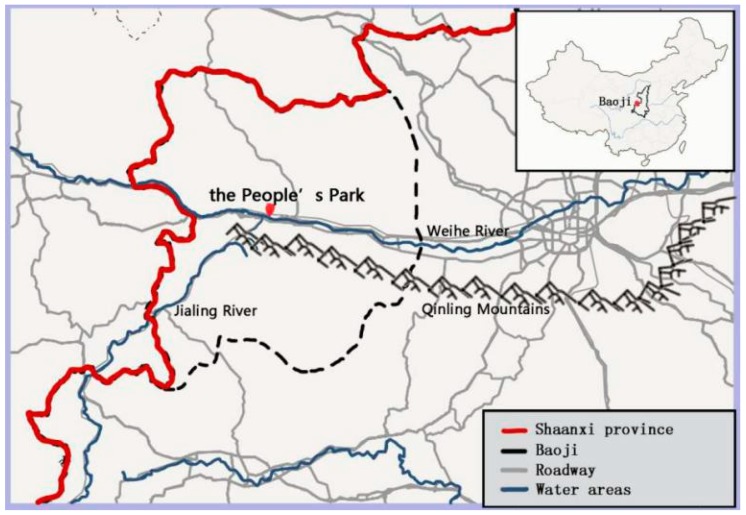
Location of the People’s Park in Baoji city near the Qinling Mountains, Weihe River and Jialing River in Shaanxi, China.

**Figure 2:**
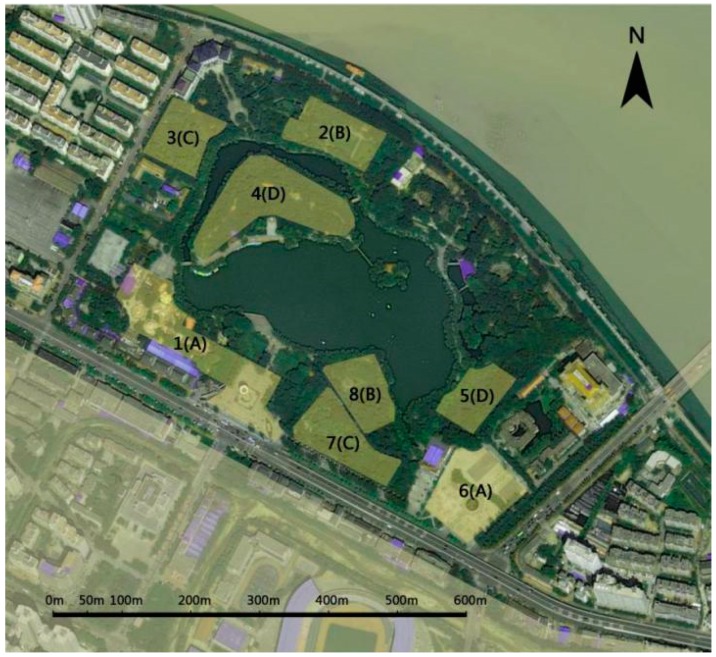
Site zoning of the People’s Park (PP) according to visual differences in landscape characteristics and vegetation structure. 1–8 represent eight visually different zones based on location, landscape attributes, composition structure and distribution characteristics of vegetation. A–D represent four different habitat types with similar characteristics but they are detached.

This study was conducted in the People’s Park (PP) in Baoji City, the second largest city of Shaanxi province, China, with a population of 3.78 million as of 2016. Baoji City is surrounded on three sides by hills, and its specific location controls a pass on the Qin Mountains between the Wei River valley and the Jialing River (Figure 1). The city won the honor of “National Garden City” in 2005 and “National Forest City” in 2009 due to its specific waterfront landscape and urban greening. The PP is a large recreational and cultural park with ancient ritual and music culture (Zhou), and it was established in 1979. In total, it covers an area of 34.6 ha with 9.3 ha of water surface. It is the largest comprehensive, multi-functional place of entertainment in Baoji that integrates leisure, physical fitness, and fauna and flora watching for city inhabitants. Today, PP attracts a large number of visitors annually, making it one of the most popular recreation areas in Baoji. In order to ensure that selected areas represent the main distinguishing features of the park, a visual and bio-physical characterisation of the landscape was conducted in August 2017 by four landscape architects and two urban ecologists. The park landscape was divided into visually distinguishable units based on field inventories and aerial photography analysis, focusing on location, landscape attributes, composition structure and distribution characteristics of vegetation. Based on the above-mentioned factors, the park was then divided into eight visually distinguishable zones. The similar but physically detached zones were clustered resulting in four habitat types (Figure 2).

Habitat A (Zone 1 and Zone 6): Grey space designed with impervious pavements and sculptures. Playgrounds for children are prominent. The landscape is a mosaic of lawn, sporadic trees and manicured shrubs. 

Habitat B (Zone 2 and Zone 8): Open green space with small sculptures. One side sets up pavilions and the other side is next to the garden road. The green space is surrounded by manicured shrubs and tall trees as background. 

Habitat C (Zone 3 and Zone 7): Closed understory green spaces with a single tree layer. A path and small paved square with recreational facilities are distributed in Zone 3 and Zone 7. 

**Figure 3:**
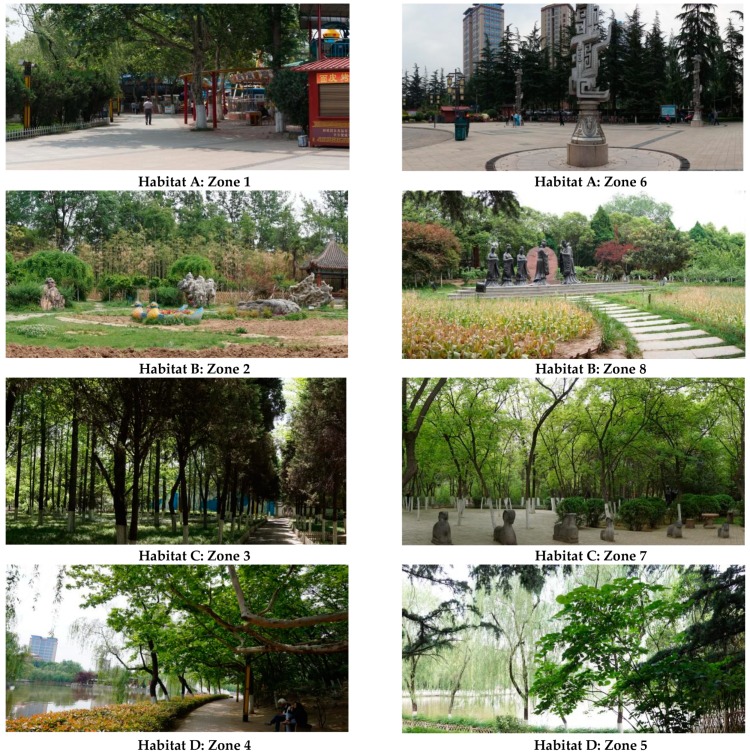
Typical landscape unit types and included regions. Habitat (**A**) grey space dominated by rigid pavement with conifer or broad-leaved evergreen trees around or at the upper boundaries; Habitat (**B**) open landscape; Habitat (**C**) single layer forest space; Habitat (**D**) Multi-layer vegetation space near the water body.

Habitat D (Zone 4 and Zone 5): Closed green space with multi-layer vegetation where at least one side is adjacent to water, which make it possible for people to appreciate the water scene. Multi-layer vegetation is flourishing and dominated by woodlands and scrubland (Figure 3).

### 2.2. Data Collection

Chinese self-reported stressed individuals’ preference for the eight sensory dimensions and recreation activities in urban green spaces was studied by means of an on-site questionnaire survey. Respondents were randomly selected among visitors to each zone at the same time regardless of their social-demographic characteristics or educational background. People approached in each zone were first informed about the survey aims and answering procedure. Those willing to participate were then given the questionnaire and were invited to fill it in during their stay in the area so that the answers would reflect their immediate experiences. Questionnaires were distributed in September, 2017, under similar weather conditions—sunny and windless or slightly windy on both weekdays and weekends at different hours of the day. The questionnaire contained four parts. The first of these contained questions revealing demographic information about the respondents including gender and age, while the second part addressed the respondents’ actual use of the site and favorite recreational activities within the whole park. The third part included questions about perceptions of, and preferences for the eight perceived sensory dimensions, which were expounded upon with informative text and illustrated with photos. For each dimension, the respondents were asked to indicate whether they perceived it within the zone. The answer included a five-point scale ranging from very weak to very strong. The respondents were also asked to rank their preferences of the eight sensory dimensions themselves by selecting from eight points to indicate their most favorite to one point indicating their least favorite in order to clarify the psychological expectations of self-reported stressed respondents for restorative environments. Finally, the respondents were asked to answer whether they preferred the specific habitat. If they preferred, they subsequently chose or filled in the activities frequently conducted there. Otherwise, they needed to fill in the reasons why they did not prefer the habitat. The last and most important part of the questionnaire contained the Level of Stress (LS) test. According to the stress study of Grahn and Stigsdotter, seven questions that are most related to the level of self-examined stress within the SCI-93 were selected. These contained “irritation,” “stress,” “fatigue,” “back ache,” “neck ache,” “common cold” and “head ache.” [6,28] Respondents were asked to choose the frequency of each condition that occurred in a year. The frequency included a total of eight scales ranging from never to daily [6].

### 2.3. Data Analysis

A total of 906 respondents completed the questionnaire, but 79 were excluded from the data set due to inconsistencies and deficiencies in their responses, resulting in a total of 827 respondents completing the survey with 166 in Habitat A, 231 in Habitat B, 224 in Habitat C and 206 in Habitat D. The respondents were divided into six age groups: <13 (1.9%), 13–17 (8.0%), 18–25 (30.4%), 26–40 (19.1%), 41–60 (21.3%) and >60 (19.3%) (Table 1). The activity list included 23 activities which were partly based on the types of activities mentioned by Huang [32], and it incorporates the special activities of the PP that were defined after field investigation, such as singing Qin Opera. The types of recreational activities were finally divided into seven types: parent-child activities (e.g., bring a child), fitness and health activities (e.g., play, tai chi and square dance), sports and leisure activities (e.g., running and walking), social activities (e.g., getting together and chatting), specialized activities (e.g., dog walking and kite flying), quiet activities (e.g., reading and sunbathing) and public participation activities (e.g., spring outing). For the level of stress, factor analysis (Varimax, orthogonal rotation) was used to test the relationship of seven stress-related questions. From this, the most relevant variable compositions for the stress level could then be determined. Finally, to treat the variables equally, principal component analysis (PCA) was used and multiplied by values to form the final formula concerning the level of stress [33,34]. The stressed individuals were subsequently grouped equally into five groups according to their stress level, in which there were the highest stress group, and the other four groups formed a new stress group that contained no to medium stress groups as a comparison.

To understand perceptions of the eight sensory dimensions between self-reported stressed user groups of urban green space in China, the study used a general linear model. Considering that the gender and age of the respondents may also affect the perception of sensory dimensions in different habitats, the study regarded the eight perceived sensory dimensions as dependent variables, and took habitat type, gender, age and stress group as fixed factors. After that, the independent sample T test within each habitat, mainly focusing on the mean differences in the perceptions of the eight sensory dimensions among the different stress groups was conducted. The perceptions of eight sensory dimensions in each habitat were considered as test variables, and the two stress groups were considered as a grouping variable.

Then, due to the unbalanced distribution of recreational activity categories and respondents with different demographic characteristics, the generalised linear model was used to examine the preference of self-reported stressed individuals for eight sensory dimensions and recreational activities. Eight sensory dimensions and seven categories of recreational activities were separately used as dependent variables, while gender, age and stress groups were used as factors. The three potential factors, i.e., gender, age and stress groups were then also separately used to test the variation tendency of preference among different sensory dimensions and recreational activity categories. To examine the preferences of the highest stress group for the eight sensory dimensions in Chinese respondents, arithmetic means and ANOVA with post hoc tests were conducted concerning the highest stress level only. 

The generalised linear model was finally conducted to identify the effects of the eight sensory dimensions on recreational activities in the selected green spaces. 

Goodness-of-fit of the models was assessed by the Pearson’s chi-squared test and deviance test, and the results indicated that the models fitted the data adequately. The statistical analyses were conducted using the statistical software package IBM SPSS 17 (SPSS Inc., Chicago, IL, USA).

## 3. Results

### 3.1. The Level of Stress and Demographic Characteristics Within Self-Reported Respondents

Among the 827 valid questionnaires, there were more men than women respondents, especially in Habitat B and Habitat C. Most of the respondents were aged 18–25, while those under 18 years old were the smallest group, totally accounting for 9.9%. By comparing the percentage of each age group among habitats, it was found that the 18–25 group was more likely to concentrate in Habitat D, followed by Habitat A, which was slightly higher than average. Respondents aged 26–60 were more evenly distributed in all habitats, while those over 60 years were more concentrated in Habitats B and C. 

**Figure 4:**
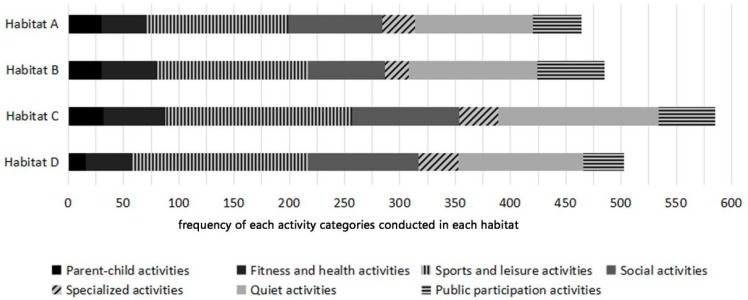
Frequency of various activity categories respondents engaged in the four habitats.

Respondents like to engage in a variety of recreational activities in the four selected habitats (Figure 4). The highest frequency of activities was in Habitat C (28.7%), followed by Habitat D and Habitat B, with the lowest in Habitat A. Sports and leisure activities and quiet activities were the most popular activity categories that respondents were willing to engage in all of these four habitats, followed by social activities, while parent-child activities and specialized activities were the least popular.

Through factor analysis for the level of stress, the study found that there were three clustered groups of factors with a total cumulative contribution of 72%. The first rotated factor consisted of stress, irritation and fatigue. The second factor consisted of back ache and neck ache, while the third factor consisted of common cold and head ache. The study by Grahn and Stigsdotter showed that stress, irritation and fatigue were significantly related to feelings of stress [28]. The values of these three in the study were all over 0.5 (stress 0.84, irritation 0.78 and fatigue 0.67), forming a strong factor, and therefore, we used these three as an indicator of level of stress (LS) expressed as

(PC stress * stress) + (PC irritation * irritation) + (PC fatigue * fatigue) = LS.

With the results of the principal component analysis of these three factors, the final formula was created, which was

(0.63 * stress) + (0.52 * irritation) + (0.58 * fatigue) =LS.

Based on the LS, respondents were divided into five grades, the highest stress group included a total of 163 participants with LS > 140 occasions per year (19.7% of the respondents), and the other four grades were collectively referred to as the “no to medium” stress group indicating respondents suffering LS < 140 per year. Highest stress respondents were found in all four types of habitats, with the highest percentage in Habitat D.

### 3.2. Perceptions of the Eight Sensory Dimensions Between Self-Reported Stressed User Groups of Urban Green Space in China

Results of the general linear model in all of the habitats showed significant differences in perception of the eight sensory dimensions (i.e. *nature*, *space*, *refuge*, *social* and *culture*, all with *p*-value < 0.05) among various age groups but seldom in the level of stress groups. However, since the level of stress with habitat had interactive significant influences on the perception of the eight sensory dimensions, the perception effects of stress levels in each habitat were then examined. Independent sample T test results showed that there were significant differences in perception of the *serene* and *prospect* sensory dimensions in Habitat D between the two stress groups (Table 2). It was found that *serene* was a more frequently experienced sensory dimension by the respondents in the highest stress group, but *prospect* was less experienced than it was by the “no to medium” stress group.

### 3.3. The Preferences of the Self-Reported Stressed Individuals for the Eight Sensory Dimensions and Specific Recreational Activities

Respondents in different age groups showed significant differences in their preferences for the perceived sensory dimensions themselves (i.e., *serene*, *space*, *prospect*, *social* and *culture*, all with *p*-value < 0.05), while gender and level of stress made significant differences to the preferences for only individual sensory dimensions, i.e., *nature* (*p* = 0.03) and *culture* (*p* = 0.01). In order to further clarify the preferences for the specific sensory dimensions of the highest-stressed respondents and test whether the mean differences between the eight sensory dimensions were statistically significant, *serene* was taken as the reference group to compare with the other seven dimensions since its arithmetic mean values of preference was highest in the highest-stressed group. Interestingly, the results showed that the highest stress respondents had significant differences in preference for the eight sensory dimensions and they most preferred the *serene* sensory dimension, followed by *space*, *nature*, *prospect*, *rich in species* and *refuge*. *Culture*, followed by *social*, had the lowest popularity (Table 3).

**Figure 5:**
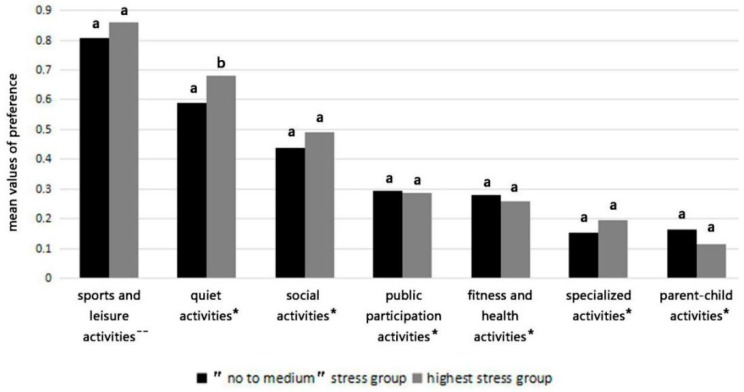
The mean values of preferences for the activity categories considering the two stress groups (no to medium stress group and highest stress group). Note I. The same letter within one activity category indicates that there is no significant difference in preference degrees for specific activity category between two stress groups, while the different letters within one activity category indicate that there is a significant difference. Note II. ^--^ represents the reference activity category with the highest mean values of preference compared with the other six activity categories. * represents that there are significant differences of the mean values of preferences for specific activity categories compared with the reference activity category.

The generalised linear model showed that level of stress had a significant effect on quiet activities (Figure 5). Respondents in the highest stress group were more likely than those in the “no to medium” stress group to engage in quiet activities. Compared with the “no to medium” stress group, the highest stress group was also more inclined to conduct sports and leisure activities and social activities, although the differences were not significant. Overall, sports and leisure activities and quiet activities were the most favoured activities, followed by social activities. Parent-child activities and specialized activities were the least preferred activities among respondents.

### 3.4. Correlations Between the Eight Sensory Dimensions and Recreational Activities

**Figure 6:**
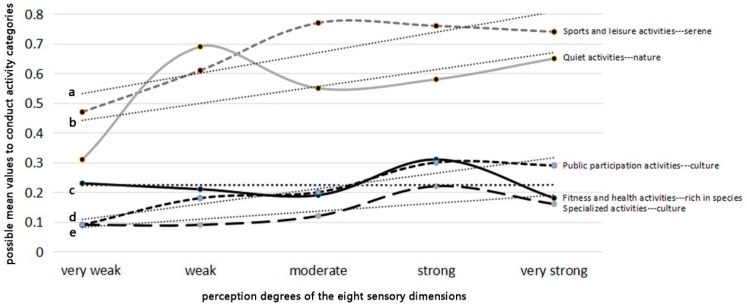
The probable mean values of conducting activity categories varies with the perceived degrees of corresponding sensory dimensions. Note I. “a” represents the trendline of sports and leisure activities. ”b” represents the trendline of quiet activities. ”c” represents the trendline of fitness and health activities. “d” represents the trendline of public participation activities. “e” represents the trendline of specialized activities.

Except for parent-child activities and social activities, each type of recreational activity was significantly related to a certain sensory dimension. For instance, the perception degree of *rich in species* sensory dimension had significant effects on the possibilities for conducting fitness and health activities (*p* = 0.02), *serene* was significant for the sports and leisure activities (*p* = 0.00), *culture* significantly related to specialized activities (*p* = 0.02) and the public participation activities (*p* = 0.03), and *nature* had significant relationships with quiet activities (*p* = 0.01). By calculating the probability mean values of conducting activity categories under different perception degrees, it could be found that with the enhancement of the perception degrees of certain sensory dimensions, the possibility to conduct corresponding activity categories mostly increased (Figure 6). For the highest-stressed respondents, green spaces with more *serene* and *nature* sensory dimensions might increase the possibilities for them to engage in sports and leisure activities and quiet activities.

## 4. Discussion

In order to search for a rich, restorative environment for human stress relief, this study explored the preferences of stressed individuals for characteristics of green spaces and relevant activities, and the potential relationships between them in Chinese urban environmental settings.

### 4.1. Perceptions of the Eight Sensory Dimensions Between Self-Reported Stressed User Groups of Urban Green Space in China

Results of the general linear model showed that age had a significant influence on the respondents’ reported experience of the *nature*, *space*, *refuge*, *social* and *culture* sensory dimensions in the four habitats. This is largely consistent with findings in another study of urban green spaces in the Chinese context [35], which concluded that the selections of recreational activities among different age groups were likely to explain these differences. Different aged groups often gravitate to different activities based on their perceptions of and preferences for a site [31]. Although the level of stress had less significant influence on the perception of the eight sensory dimensions in all of the habitats, it still showed a significant difference in Habitat D. Compared with the “no to medium” stress group, the highest stressed group had more experience of the *serene* sensory dimension and less of the *prospect* in Habitat D. The specific characteristics of the site are likely to explain these differences. Sang et al. claimed that natural green spaces had greater potential to provide more opportunities for perceptual experience [36]. The openness and composition of the ornamental park, the views over the water and the complex structure of the woodlands were frequently perceived as positive settings of natural elements. This is in line with Wilkie and Stavridou, who found high levels of visual landscape complexity to be often appreciated by people [37]. Habitat D is a multi-layered woodland near a water body with few artificial recreational facilities, which is totally different compared to the other habitats (A to C) that are common green spaces sporadically distributed among urban areas in China. Current Chinese urban landscape patterns have usually led to less *serene* experiences in city environments [38]. Chinese inhabitants, especially the highest stressed respondents, are eager to escape from the city to urban forests to seek calmness and tranquillity instead of the bustle of urban life [39]. The rich vegetation and views from the water body might be conducive to controlling their emotions and enjoying them, thus they experience serene more frequently. ‘Quietness,’ as the label of *serene* [40] is considered to be one of the main reasons people come to a green space [41], which can be explained by psychological stress relief with the contribution from noise exposure during the visits [28]. On the other hand, Chinese urban inhabitants are often surrounded by urban squares and affiliated small green spaces close to their living places [35], where *prospect* is more easily perceived due to the open views created by artificial pruning and high intensity of management. Such habitual experiences of ‘*prospect*’ will not help urban inhabitants to relieve their stress as compared to a more natural environment. This is why there was the largest number of highest-stressed respondents visiting Habitat D in this study. This indicated that respondents had certain cognitive abilities in the green spaces [42], especially for the highest-stressed respondents, who were more inclined to an environment with more *serene* and less *prospect* experiences.

### 4.2. The Preferences of the Self-Reported Stressed Individuals for the Eight Sensory Dimensions and Specific Recreational Activities

Different aged respondents had significant differences in their preferences for the *serene*, *space*, *prospect*, *social* and *culture* sensory dimensions themselves, which implied that different age groups had different purposes for visiting green spaces and naturally required the corresponding characteristics of the green spaces to meet their purposes. McGinlay et al. and Coldwell and Evans both found that nature-oriented activity selections and high frequency use of green spaces affect people’s environmental awareness and knowledge, and probably preference [43,44]. Gender groups had significant differences on preference of *nature*, which could be explained due to their different cognition of nature. Stafford et al. found that women have greater exposure to their neighbourhood environment and are more vulnerable to its health effects than men [45]. Levels of stress were significantly associated with *culture* preference. People under the highest stress could have a lower preference for *culture*. This may be attributed to the ubiquitous man-made sculptures and facilities sporadically distributed in the park, which led to the asthenopia and negative effect of attention restoration [35].

Highest-stressed respondents preferred *serene* most, followed by *space* and *nature*. *Prospect*, *rich in species* and *refuge* were preferred next, while *culture* and *social* were the least preferred. This indicates that the highest-stressed respondents need to visit green spaces on a more fundamental level with less human intervention and more opportunities to increase emotional and physical engagement [27]. Since the preference for a place is the result of multiple sensory interactions, *serene*, which does not mean absolute silence but no noise, can be achieved by birdsong or other natural elements combined [27]. This natural serenity often works as a good experience for relaxing and mental health related needs and is associated with large areas of urban green space. Large green space may be rare in urban cities, but it is easy to change the cityscape to create an urban forest atmosphere. This is more popular due to the option to link more sensory dimensions [35] and it is beneficial to stress relief [46]. *Space* and *nature* are also positively correlated to large areas of urban green space. Accessibility, as an influencing factor on human health [47,48] brings significant convenience for experiencing nature. Urban inhabitants can be free to walk and do some activities in natural environments with a certain degree of accessibility and connectivity (*space*) [27]. There can even be tall trees and terrain changes to increase the attractiveness of the green space [49]. Wild and luxuriant plants can promote the sense of *nature*, which can usually be found away from the downtown. All of these attributes potentially increase the compatibility of the green space, which is closely related to stress recovery. People instinctively and positively react to the natural environment (e.g., [50,51]) and this might be helpful in improving people’s self-perceived health. The low level of *culture* preference may be related to the fact that the cultural atmosphere is prominent through artificial facilities rather than natural elements in the PP, and this experience is not conducive to stress recovery. A *social* dimension is widely perceived in various types of green spaces in the PP. It may focus more on social interaction to promote green space visitation and then stress recovery. However, people under the highest level of stress tend to be intolerant of others [52], and they lack of interest in social interaction experiences. The low preference for the *social* dimension among highest-stressed respondents may be due to the high burden of population agglomeration.

This study clearly identified the gradient of preferences of the highest stressed respondents for the eight sensory dimensions themselves rather than in actual green spaces, in order to clarify their psychological expectations of a restorative environment. It was found that the psychological expectations of the highest stressed respondents were generally consistent with their actual perceptions in the preferred habitat (Habitat D), i.e., an environment with more *serene* experiences and less *prospect*. In addition, although there were no significant differences in preference for *culture* and *social* among people with different levels of stress in Habitat D, the highest stressed respondents still perceived less *culture* and *social* there. Therefore, an environment with more *serene*, less *prospect* and that avoids *social* and *culture* dimensions is extremely desired by self-reported stressed people. 

Regardless of age and gender, stress level had a significant influence on the preference for quiet activities, which had also shown the higher mean values of preference in the highest stress group. Searles claimed that people who face more difficulties in life were more likely to revert to simpler situations due to the convenience of handling simpler situations [53]. As to highly stressed respondents, the natural environment is often accompanied by less loss of attention [54] and more emotional regulation [15], and can certainly offer opportunities for them to engage in quiet activities such as reading, sunbathing, meditation and relaxing, etc. Feelings of calmness and perceived benefits for mental health from such activities could then explain the higher level of preference. Apart from quiet activities, it was found that sports and leisure activities and social activities were popular with the highest-stressed respondents, while parent-child activities and specialized activities were less appreciated. That is because people often use urban parks predominantly for engaging in physical activities with some variation in social park uses, depending on their motives [49]. Sports and leisure activities and social activities can provide opportunities to reduce loneliness and increase social cohesion, which work as a health boosting mechanism of green space and leads to more satisfaction from the highest-stressed respondents. Compared with the popular activities mentioned above, parent-child activities and specialized activities were conducted more in the selected green spaces with specific facilities (e.g., pavilions for Qin opera, dog paths for walking with dogs) by specific demographic populations, which may have led to the lower preference considering the overall respondents. Accordingly, a specific site could be designed for the different perceived-stressed groups to engage in the recreational activities that they gravitate to based on their perceptions and preferences.

### 4.3. Correlations Between the Eight Sensory Dimensions and Recreational Activities

The study found that self-reported stressed individuals preferred to conduct sports and leisure activities and quiet activities in green spaces with *serene* and *nature* sensory dimensions, specialized activities and public participation activities with *culture*, and fitness and health activities with a strong perception of *rich in species*. Previous studies showed that the physical existence of green space and people’s perception affect the way people use green space [55,56]. The significant relationships between the perception of *culture* and conduction of specialized activities and public participation activities were possibly due to the cultural theme of the PP. These activity categories reflected the culture of the park and acted as the representative activities in this park, while for fitness and health activities, higher species richness might give the space a sense of encirclement and at the same time increase shade. Self-reported stressed individuals preferred to conduct activities in the green space with more *serene* and *nature*. *Serene* and *nature* can not only satisfy their preferences for and expectation of the green space attributes, but also grant the opportunity for sports and leisure activities and quiet activities, which have been found to be the most popular and the most frequently conducted activities among self-reported stressed individuals. Quiet activities here were closely related to sunlight, grass and tall trees, which commonly reflect the wildness and forces of nature. The lush vegetation may create a safe and private environment that provides a potential space to relax and meditate. It was found that respondents were more likely to go running, walking, visiting and do mental relaxation in quiet and forest-like spaces, for instance, in Habitat C and Habitat D, where there was calmness with tall trees and various plant species. Due to the high habitat-dependence and the low frequency of conducting fitness and health activities, specialized activities and public participation activities, the enhancement of sports and leisure activities and quiet activities will have significant and collective implications for the improvement of restorative environmental quality. Accordingly, for future restorative environment design, sites should be created with more *serene* and *nature* experiences to appeal to self-reported stressed people so that they can have more opportunities to engage in sports and leisure activities and quiet activities.

## 5. Conclusions

This paper is the first to explore the perceptions of and preferences for certain characteristics of urban green spaces represented by eight sensory dimensions and activities within self-reported stressed individuals in Chinese green spaces. It also aims to provide suggestions for a restorative environment design for human stress relief. The relationship between the eight sensory dimensions and recreational activities have also been examined. There are some noteworthy conclusions. First, highest-stressed respondents tended to gather in the multi-layer vegetation spaces by the water, where they experienced a more *serene* sensory dimension and less *prospect*. Second, highest-stressed respondents preferred *serene* most, followed by *space* and *nature*, while they preferred *culture* and *social* least. The results indicated that the expected attributes of an environment for the highest-stressed people were consistent with their perceived attributes. Sports and leisure activities and quiet activities were the most popular activity categories, followed by social activities. Finally, there were close relationships between recreational activities and the eight perceived sensory dimensions. Fitness and health activities, sports and leisure activities and quiet activities were significantly associated with *rich in species*, *serene* and *nature*, respectively. Specialized activities and public participation activities were significantly associated with *culture*. Overall, stress restoration for urban inhabitants can be achieved by improving green space qualities during the planning and management process. Green spaces with more *serene* and *nature* sensory dimensions, less *prospect* and few or no *culture* and *social* dimensions are regarded as the most restorative places for self-reported stressed people to conduct physical activities for stress relief. These conclusions have crucial implications for enhancing the capacity of green space to address mental health. For instance, urban green spaces with more *serene* and less *prospect* can potentially increase the concentration and appreciation of the highest-stressed individuals, while green spaces with more *social* and *culture* might repel them. By increasing the experience of *serene* and *nature* in green spaces, the perceived stress respondents can increase their opportunities to engage in sports and leisure activities and quiet activities that are positively related to stress recovery. 

However, there are some weaknesses in this study. First, the methodological tool of the eight sensory dimensions was developed in Scandinavia, and due to the variations in personal experiences that are partially influenced and learned through socialisation, cultural and geographical contexts, a full understanding of the extent to which those same perceived dimensions of relevance for Scandinavian population can be applied effectively to measure the restorative experience elsewhere is needed [57]. Second, this study was confined to one park and a limited number of habitats, which may be considered an oversight but therefore make it difficult to obtain universal conclusions. Third, the study found some certain significant effects of demographics on the perceptions and preferences of self-reported stressed people, but these were not discussed in detail in this study. Moreover, self-reported stressed respondents with different social characteristics tended to conduct different activities in the green spaces at different times [58,59,60]. Therefore, in order to identify the best restorative environment for human stress relief in China, further investigations within different contexts, combined with more sociodemographic and spatiotemporal characteristics of self-reported stressed participants, should be examined.

## Figures and Tables

**Table 1:** The demographic information considering gender and age of the respondents in the PP (People’s Park).

	Habitat A	Habitat B	Habitat C	Habitat D	Sum	Percentage
Gender						
Men	83	148	140	102	473	57.2%
Women	83	83	84	104	354	42.8%
Age						
<13	2	3	9	2	16	1.9%
13–17	16	19	9	22	66	8.0%
18–25	61	42	50	98	251	30.4%
26–40	37	41	48	32	158	19.1%
41–60	28	57	59	32	176	21.3%
>60	22	69	49	20	160	19.3%
Sum	166	231	224	206	827	100.0%

**Table 2:** Results from independent sample T test between the impacts of two stress level groups on the perception of eight sensory dimensions in each habitat.

**Habitat**	**Serene**		**Nature**		**Rich in species**		**Space**	
	**MD** ^1^	**95% C.I.**	**MD** ^1^	**95% C.I.**	**MD** ^1^	**95% C.I.**	**MD** ^1^	**95% C.I.**
A	0.22	−0.25–0.69	0.23	−0.22–0.67	0.28	−0.16–0.73	−0.19	−0.56–0.18
B	−0.20	−0.54–0.14	0.07	−0.26–0.41	−0.08	−0.40–0.24	−0.16	−0.49–0.18
C	0.09	−0.24–0.43	−0.08	−0.39–0.24	−0.13	−0.45–0.20	0.23	−0.09–0.54
D	−0.28 *	−0.55–−0.02	0.23	−0.04–0.49	0.22	−0.10–0.54	0.06	−0.22–0.35
**Habitat**	**Prospect**		**Refuge**		**Social**		**Culture**	
	**MD** ^1^	**95% C.I.**	**MD** ^1^	**95% C.I.**	**MD** ^1^	**95% C.I.**	**MD** ^1^	**95% C.I.**
A	−0.12	−0.54–0.30	0.07	−0.32–0.46	0.07	−0.30–0.45	−0.05	−0.48–0.38
B	−0.18	−0.56–0.20	0.04	−0.31–0.39	−0.30	−0.66–0.06	−0.14	−0.54–0.26
C	−0.02	−0.37–0.33	0.10	−0.23–0.42	0.07	−0.29–0.43	−0.18	−0.57–0.20
D	0.49 **	0.17–0.82	0.01	−0.28–0.30	0.06	−0.30–0.41	0.20	−0.17–0.57

^1^ MD represents the difference of perception mean values between “no to medium” stress group and highest stress group. * The significant level of mean difference is 0.05. ** The significant level of mean difference is 0.01.

**Table 3:** Arithmetic mean value and post hoc tests in ANOVA concerning the preferences of respondents in highest stress group for eight sensory dimensions (N = 163).

Perceived Sensory Dimensions	Arithmetic Mean Values			ANOVA with Post Hoc		Rank
	Mean	SD	SE Mean	Mean Difference ^1^	95% C.I.	
Serene	6.52	1.64	0.13	Reference	—	1
Space	5.73	1.80	0.14	0.79 **	0.37–1.21	2
Nature	5.43	2.04	0.16	1.09 **	0.67–1.51	3
Prospect	4.94	2.03	0.16	1.58 **	1.16–2.00	4
Rich in species	3.90	2.14	0.17	2.62 **	2.21–3.04	5
Refuge	3.53	1.75	0.14	2.99 **	2.57–3.41	6
Culture	3.32	1.81	0.14	3.20 **	2.78–3.62	7
Social	2.72	2.14	0.17	3.80 **	3.39–4.22	8

^1^ Mean difference means the difference of preference mean values between *serene* and other seven sensory dimensions. ** The significance of mean difference is at the 0.01 level.
